# Disruption of sleep macro- and microstructure in Alzheimer’s disease: overlaps between neuropsychology, neurophysiology, and neuroimaging

**DOI:** 10.1007/s11357-024-01357-z

**Published:** 2024-09-28

**Authors:** Anna Csilla Kegyes-Brassai, Robert Pierson-Bartel, Gergo Bolla, Anita Kamondi, Andras Attila Horvath

**Affiliations:** 1 School of PhD Studies, Semmelweis University, Budapest, Hungary; 2 Faculty of Medicine, Semmelweis University, Budapest, Hungary; 3 Neurocognitive Research Centre, Nyírő Gyula National Institute of Psychiatry, and Addictology, Budapest, Hungary; 4 Department of Neurosurgery and Neurointervention, Semmelweis University, Budapest, Hungary; 5 Department of Neurology, Semmelweis University, Budapest, Hungary; 6 Department of Anatomy Histology and Embryology, Semmelweis University, Budapest, Hungary; 7 HUN-REN Research Centre for Natural Sciences, Budapest, Hungary

**Keywords:** Alzheimer’s disease, Polysomnography, Sleep, K-complex, Cingulate cortex

## Abstract

Alzheimer’s disease (AD) is the leading cause of dementia, often associated with impaired sleep quality and disorganized sleep structure. This study aimed to characterize changes in sleep macrostructure and K-complex density in AD, in relation to neuropsychological performance and brain structural changes. We enrolled 30 AD and 30 healthy control participants, conducting neuropsychological exams, brain MRI, and one-night polysomnography. AD patients had significantly reduced total sleep time (TST), sleep efficiency, and relative durations of non-rapid eye movement (NREM) stages 2 (S2), 3 (S3), and rapid eye movement (REM) sleep (*p* < 0.01). K-complex (KC) density during the entire sleep period and S2 (*p* < 0.001) was significantly decreased in AD. We found strong correlations between global cognitive performance and relative S3 (*p* < 0.001; *r* = 0.86) and REM durations (*p* < 0.001; *r* = 0.87). TST and NREM stage 1 (S1) durations showed a moderate negative correlation with amygdaloid and hippocampal volumes (*p* < 0.02; *r* = 0.51–0.55), while S3 and REM sleep had a moderate positive correlation with cingulate cortex volume (*p* < 0.02; *r* = 0.45–0.61). KC density strongly correlated with global cognitive function (*p* < 0.001; *r* = 0.66) and the thickness of the anterior cingulate cortex (*p* < 0.05; *r* = 0.45–0.47). Our results indicate significant sleep organization changes in AD, paralleling cognitive decline. Decreased slow wave sleep and KCs are strongly associated with cingulate cortex atrophy. Since sleep changes are prominent in early AD, they may serve as prognostic markers or therapeutic targets.

## Introduction

Alzheimer’s disease (AD) is a progressive neurodegenerative disorder leading to cognitive decline and memory loss. AD is the most common cause of dementia in older adults, accounting for 60–80% of all dementia cases. Noticeably, 75% of all dementia cases are not diagnosed globally [1]. AD is a significant public health problem, with an estimated 50 million people worldwide living with the condition. Due to the aging population, this number could rise to 153 million by 2050 [2]. AD progresses through several stages as preclinical AD, mild cognitive impairment (MCI), and AD dementia. According to the established scientific viewpoint, genetic risk factors and environmental factors are both important in the early changes of the neural system, resulting in the misfolding of two physiological proteins such as amyloid and tau [3]. The toxic oligomers of amyloid and tau proteins generate a cascade leading to neural loss and consecutive cognitive complaints [4]. Currently, there are only a few FDA-approved medications [5] designed to address the cognitive and behavioural symptoms of the disease; however, these medications do not modify significantly the disease progression. Although some disease-modifying treatments became also available recently, their effectiveness and safety remain insufficiently confirmed [6]. For this reason, early diagnosis and proper recognition of potentially modifiable comorbid conditions are crucial steps to treat patients effectively.

Investigation of sleep as a comorbid alteration in AD is intriguing, since impaired sleep quality is a well-known symptom of AD. Previous reports systematically found that at least 15% of people with AD suffer from severe sleep problems [7]. A recent meta-analysis demonstrated that people with AD show significant alteration of sleep macrostructure including the reduction of total sleep, REM, and slow-wave sleep (SWS) duration and increase of sleep onset time and REM latency [7]. Since these changes are very robust and frequent, they might be potential candidates for biomarkers helping the early detection, indicating the progression or therapeutic responses in AD [8]. Some studies have suggested that positive amyloid profiles—which are the most characteristic pathologic hallmark of AD—are associated with lower subjective sleep quality and longer awake time spent in bed, irrespective of cognitive status [9]. Since amyloid misfolding starts decades before the first onset of cognitive symptoms [10], these findings suggest the utility of sleep macrostructure as screening or diagnostic markers [11]. On the other hand, disruption of the circadian rhythm and fragmentation of sleep can accelerate the progression of AD, since under normal conditions, sleep facilitates the removal of amyloid beta (Aβ) from the neural tissue [12, 13]. These results imply that there is a positive feedback loop between amyloid deposition and the alteration of sleep [14], suggesting that improving sleep may have therapeutic potential in the treatment of AD. Thus, analysis of sleep alterations could assist in the monitoring of disease progression [15] and therapeutic responses [16]. Unfortunately, limited knowledge exists on the interrelation of structural brain changes related to altered sleep macrostructure in AD, compromising the ability for deeper understanding and biomarker utility [7].

Novel observations also found significant reorganization of sleep microstructure with the alteration of sleep spindle and K-complex (KC) density [17]. Sleep microstructure refers to the short and rapid changes in sleep patterns that occur throughout the night, such as sleep spindles, KCs, and slow-wave activity (SWA). Sleep spindles are burstlike signals on the electroencephalography (EEG) in the mammalian brain, generated in the thalamo-cortical networks [18]. Occurrence of spindles represents a crucial state of memory consolidation where a high synchronicity is presented in the hippocampo-thalamo-cortical circuits [19]. This stage provides an ideal window for the shift between temporally generated hippocampal synaptic connections (short-term memory) and the permanently formed cortical synaptic connections (long-term memory), known as the two-stage memory consolidation model [20, 21]. Neocortex in also involved in the process with the generation of subsequent SWA as a default mode neural activation in the high-order cortical networks [22]. The generated SWA serves as a driver for the repeated reactivation of the newly encoded hippocampal information [23] which also facilitates the transformation of the recent information to the neocortex [24]. The reduction of SWA [7, 25] and the diminishment of sleep spindles [26] are constant findings in Alzheimer literature. Furthermore, growing number of studies propose their utility as a therapeutic target and biomarker [27, 28].

While a stable literature background is available on SWA and sleep spindles related to AD, the number of studies on KC is relatively limited and results are frequently conflicting. KC is often seen as the largest graphoelement in SWS throughout the night [29]. KC is a high-amplitude biphasic wave described by a surface-negative wave (down-state) with neural hyperpolarization, followed by a positive wave (up-state) during which neurons are depolarized [30]. Nevertheless, the origin of KCs in the mammalian brain is still under investigation [31], and its paradoxical character also generated scientific debates on its primary role as an arousal sign or sleep protective phenomenon [32]. KCs could be generated by external stimuli (evoked potential) and could occur spontaneously [33]. Besides sleep spindles, KC is also a regular component of SWS showing similar aging pattern, namely a characteristic reduction of their density with aging [34]. Some of the intracranial landmark studies helped us to describe the most characteristic features of KC: (1) they represent a cortical down state with a hyperpolarized state of the generator neurons [35]; (2) KCs do not propagate in a systematic way and can co-occur in small and large cortical regions as well [36]. Since KC represents one of the most synchronized down-state activities of cortical neurons, it can be used to monitor the integrity of the central nervous system and applied as a biomarker for disease monitoring [31].

From the early 2000s, researchers made the paradoxical observation of increased SWA and decreased KC density in people with AD compared to healthy elderly individuals [37, 38]. This may be explained by the partial overlap between 0.6 and 1 Hz EEG activity and KCs leading to the question: Can KC density and amplitude be sensitive biomarkers for early diagnosis in people with AD or MCI? Some groups have found negative results [39] and argue that no differentiation can be made between people with MCI and healthy individuals, while others have found a steeper decrease in KC density in people with MCI over a 2-year follow-up period [40], also taking overall sleep architecture differences into account [41]. Analysing the cortical mechanisms of KC generation might answer the proposed uncertainties.

In the current study, we aim to characterize the macro- and microstructural differences between healthy individuals and people with AD. We also address the underlying causes of the potential differences by analysing the relation of sleep characteristics—neuropsychological profile and neuroimaging data. The major clinical endpoint is to examine the potential utility of sleep characteristics as a diagnostic, disease progression, or therapeutic biomarker in AD.

## Methods

### Participants and clinical testing

The examinations took place at the Department of Neurology at Semmelweis University Department of Neurosurgery and Neurointervention (previously known as National Institute of Mental Health, Neurology, and Neurosurgery) in Budapest, Hungary. All participants underwent an extensive dementia screening protocol including neurological, imaging and laboratory examinations with thyroid functions, vitamin B12, and folate levels. The AD diagnosis was given based on the guidelines of the National Institute on Aging and the Alzheimer’s Association [42]. The healthy control participants had intact cognitive status based on neuropsychology, negative neurological status, and no clinically significant atrophy or brain lesions assessed with brain MRI. Subjects with known risk factors of cognitive decline or sleep disorders were excluded in line with the following criteria: untreated vitamin B12 deficiency, untreated hypothyroidism, liver disease, renal insufficiency, sleep-related breathing disorders, alcohol abuse, substance abuse, use of psychoactive drugs influencing cognitive function except for anti-dementia medication, use of benzodiazepines, clinically significant structural brain lesions, earlier head injury with loss of consciousness, HIV infection, schizophrenia, electroconvulsive therapy, major depression, syphilis, or other prior central nervous system infections. Overall, 30 participants with AD diagnosis and 30 healthy control participants were involved in our study. All experimental protocols were approved by The Hungarian Medical Research Council (reference number of ethical approval: 024505/2015). We obtained informed written consent from each participant. All methods were carried out in accordance with relevant guidelines and regulations.

### Neuropsychology

Neuropsychological tests were carried out by qualified neurologists and neuropsychologists in Hungarian. We selected the Hungarian version of the Addenbrooke Cognitive Examination (ACE) to assess global cognitive functioning which includes six modules: orientation, attention/concentration, memory, verbal fluency, language, and visuospatial abilities. The maximum score of modules were 10, 8, 35, 14, 28, and 5, respectively resulting a total score of 100 [43]. Anxiety and depression were examined with the Spielberger State and Trait Anxiety Inventory (STAI) and Beck Depression Inventory II (BDI-II). To reduce the influence of anxiety and depression on the results, participants with a BDI-II score of > 13 or a STAI score of > 45 were not included in this study [44].

### Neurophysiology

For sleep recording, a 34-channel 24-h-long electroencephalogram (EEG) (Micromed Morpheus Holter EEG) was performed. The electrodes were placed according to the 10–20 system. The electrode labelling was the following: frontopolar (Fp1, Fp2), frontal (F3, F4), frontocentral (Fz) temporal (T3, T4, T5, T6), central (C3, Cz, C4), centroparietal (Pz), frontotemporal (F7, F8) parietal (P3, P4), and occipital (O1, O2). Electrooculogram (EOG), electromyogram (EMG), and electrocardiogram (ECG) were also registered. The electrodes were fitted by a qualified technician. The data procession was carried out using Hume, an open-source sleep analyser Matlab toolbox (https://www.jaredsaletin.org/hume). After manually removing the artefacts and defining 30-secundum long epochs in bipolar montage, data integrity and staging quality were reinforced visually by sleep research experts, guided by the American Academy of Sleep Medicine (AASM) Manual for the Scoring of Sleep and Associated Events version 2.4, 2017. During the staging process, rapid eye movement (REM), S1 (first stage of non-rapid eye movement sleep: NREM), S2 (second stage of NREM), S3 (third stage of NREM), and wakefulness phases were identified. As a result of the staging process, a hypnogram was created [45, 46]. Overall sleep quality was evaluated by a qualified neurologist. For the microstructural sleep analysis, KCs were visually identified based on the following criteria: (1) well-delineated, negative sharp wave, immediately followed by a positive component; (2) duration is between 0.5 and 3 s; (3) with maximum amplitude at the frontocentral channels [38]. Different variables were calculated including sleep efficiency (time spent in bed divided by TST), sleep latency (time from going to bed to falling asleep), relative duration of different sleep stages (percentage of the time spent in that stage compared to the TST; S1, S2, S3, and REM), REM latency (time from sleep onset to the first REM period), and KC densities (number of KCs divided by the length of S2 stage, number of KCs divided by TST).

### Magnetic resonance imaging (MRI) data acquisition and analysis

All participants underwent a whole brain MRI examination using a Siemens Magnetom Verio 3 T machine (Siemens Healthcare, Erlangen, Germany) using a standard 12-channel head receiver head coil. To check for possible pathological lesions, T2-weighted, diffusion—and a FLAIR—sequences were used. The imaging protocol also included a T1-weighted 3D MPRAGE (magnetization prepared rapid gradient echo) anatomical imaging (TR = 2300 ms; TE = 3.4 ms; TI = 1100 ms; flip angle = 12°; voxel size: 1.0 × 1.0 × 1.0 mm). Cortical reconstruction and volumetric segmentation were carried out with the freely available Freesurfer 6.0 software (http://surfer.nmr.mgh.harvard.edu). Cortical volume (mm^3^) and cortical thickness were measured from several cortical regions. The entire data acquisition process is detailed in previous articles [47, 48].

### Data analysis

IBM SPSS 20 was used for the statistical analysis. Data distribution was tested with the Shapiro–Wilk test. Epidemiological characteristics were compared with independent sample two-tailed *t* test, Chi-square test, or Wilcoxon test. Group differences were demonstrated with mean/standard deviations in normal distribution and median/interquartile ranges (IQ1, IQ3) in non-parametric distribution. The group comparison in sleep variables including total sleep time (TST), sleep efficiency, sleep latency, relative duration of different sleep stages, REM latency, and KC densities were compared with two-tailed, independent samples *t* test using age and sex as covariates. Bonferroni correction was applied for multiple comparisons. For correlational analysis due to the nonparametric data distribution, Spearman’s correlation was used. Correlation analysis was carried out between ACE score and sleep macrostructure data (e.g., TST, sleep efficiency, relative S1-, S2-, S3-, and relative REM duration, and REM latency), also between ACE subscores (e.g., orientation, attention/concentration, memory, verbal fluency, language, and visuospatial skills) and sleep macrostructure data. Further correlation was calculated between the thickness/volume of different brain regions and the sleep macro- and microstructure parameters, as well as between ACE score and sleep microstructure data (e.g., KC density averaged for TST, KC density averaged for S2 sleep duration). Statistical significance level was set at *p* < 0.05. Effect size was measured in Cohen’s *d*, where the following parameters served as benchmarks: 0.2 small effect, 0.5 medium effect, and 0.8 large effect.

## Results

### Characteristics of the measured groups

In total, 60 individuals (21 male and 39 female) participated in this study (Table 1). The mean age was 74 ± 8.8 years. Thirty participants (11 male, 19 female) were diagnosed with clinically defined AD (Group 1), and 30 participants (10 male, 20 female) were healthy controls (Group 2). The AD group was significantly older; however, the effect size was small. Due to the inclusion criteria, patients had significantly reduced values in global cognitive scores. Based on the neuropsychological profile, all people with AD had mild-moderate stage of dementia.

**Table 1:** Clinical and epidemiological characteristics of the study groups

Parameter	AD	HC	*p* value	Effect size
Participants (*n*)	30	30	-	-
^a^Female, *n* (%)	19 (63.33%)	20 (66.67%)	0.927	0.02
^b^Age (years), mean ± SD	74.2 ± 11.56	73.83 ± 5.05	0.01*	0.04
^b^ACE total score, mean ± SD	59.13 ± 11.68	94.2 ± 4.62	< 0.001*	3.94
^c^Education (years), median ratio (IQ1–Q3)	17.0 (12.0–17.0)	17.0 (12.0–17.0)	1	0.01
Age at disease onset (years), mean ± SD	69.6 ± 11.21	-	-	-
Disease duration (years), median ratio (IQ1–Q3)	4.0 (2.25–5.75)	-	-	-

*AD* group of people with AD, *HC* healthy controls, *SD* standard deviation, *ACE* Addenbrooke Cognitive Examination, *IQ* interquartile range

^a^Indexes Chi-square test

^b^Indexes *t* test

^c^Indexes Wilcoxon test

*Indicates statistically significant differences (*p* < 0.05)

### Group differences in sleep macro- and microstructure

Significant differences were found in several sleep parameters between people with AD and HCs (Table 2). People with AD had significantly longer duration of S1 (*F* = 285.65; *p* < 0.001), shorter duration of S2 (*F* = 150.35; *p* < 0.001) and S3 (*F* = 32.02; *p* < 0.001), and decreased sleep efficiency (*F* = 41.48; *p* < 0.001). The most significant differences in sleep microstructural parameters were in KC density averaged for S2 sleep duration, which was highly reduced in the AD group (*F* = 0.39; *p* < 0.001). Sex did not have a significant modifier effect (*p* = 0.31), while increased age appeared to associate with significantly longer superficial sleep and reduced REM and deep sleep (*p* values < 0.001). However, K complex density was not affected by sex and age factors (*p* values > 0.05).

**Table 2:** Group differences across the study groups

Parameter	AD	HC	*F* value	Nominal *p* value	Effect size
^a^Total sleep time (min), mean ± SD	301.41 ± 83.72	365.54 ± 48.87	13.13	0.001*	0.935566
^a^Sleep efficiency (%), mean ± SD	58.2 ± 14.65	78.73 ± 9.49	41.48	< 0.001*	5.907154
^a^Sleep latency (min), mean ± SD	39.52 ± 39.49	15.36 ± 8.12	10.78	0.002*	0.847486
^a^Relative S1 duration (%), mean ± SD	46.81 ± 12.36	7.58 ± 2.98	285.65	< 0.001*	4.363605
^a^Relative S2 duration (%), mean ± SD	25.45 ± 8.98	52.19 ± 7.88	150.35	< 0.001*	3.165275
^a^Relative S3 duration (%), mean ± SD	9.33 ± 4.93	18.72 ± 7.64	32.02	< 0.001*	1.460477
^a^Relative REM duration (%), mean ± SD	14.79 ± 9.57	21.51 ± 10.44	6.76	0.012	0.67103
^a^REM latency (min), mean ± SD	109.67 ± 64.81	108.74 ± 62.26	0.003	0.96	0.014635
^a^Number of sleep cycles (REM periods), mean ± SD	3.47 ± 1.36	3.6 ± 0.97	0.192	0.66	0.110057
^a^K-complex density (n/total sleep time), mean ± SD	0.484 ± 0.081	0.609 ± 0.096	0.22	< 0.001*	1.407385
^a^K-complex density (n/relative S2 duration), mean ± SD	0.5 ± 0.077	0.782 ± 0.091	0.39	< 0.001*	3.345546

Abbreviations *AD* group of people with AD, *HC* healthy controls, *SD* standard deviation, *REM* rapid eye movements

^a^Indexes age and sex weighted independent sample two-tailed *t* test

*Indicates statistically significant differences as *p* was set at 0.0045 applying Bonferroni correction for multiple comparisons

### Relationship between neuropsychological parameters and sleep macrostructure alterations

**Fig. 1:**
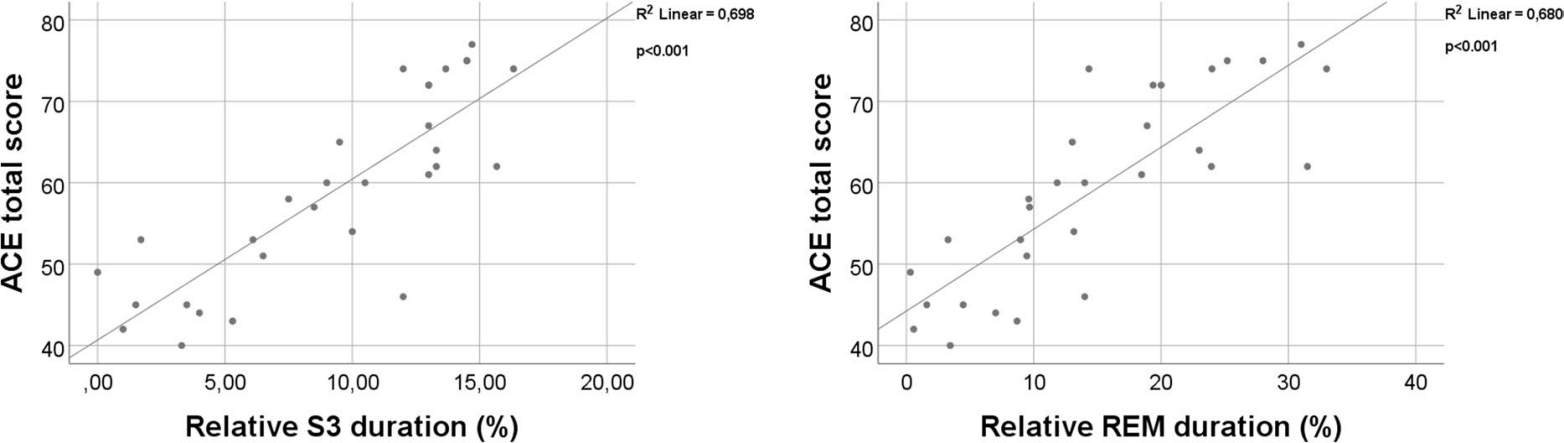
Correlation analysis between ACE total score and sleep macrostructure parameters using Spearman correlation. Key: ACE Addenbrooke Cognitive Examination, REM rapid eye movements, S3 third stage of NREM

**Fig. 2:**
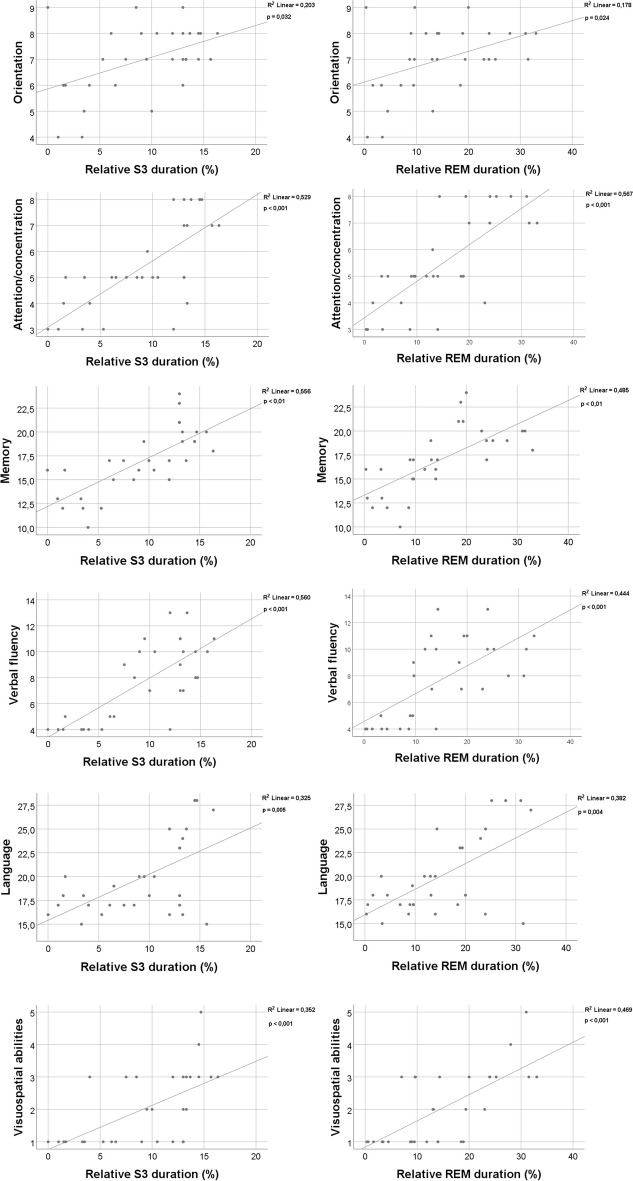
Correlation analysis between ACE subscores and sleep macrostructure parameters using Spearman correlation. Key: ACE Addenbrooke Cognitive Examination, REM rapid eye movements, S3 third stage of NREM

The ACE total score was higher in subjects with longer relative S3 (*p* < 0.001; *r* = 0.86) and REM (*p* < 0.001; *r* = 0.87) sleep duration. As for ACE subscores, patients with higher orientation scores spent more time in relative S3 duration (*p* = 0.032; *r* = 0.39), and relative REM duration (*p* = 0.024; *r* = 0.41), with higher attention/concentration score, spent more time in relative S3 duration (*p* < 0.001; *r* = 0.74) and relative REM duration (*p* < 0.001; *r* = 0.76), with higher memory scores spent more time in relative S3 duration (*p* < 0.01; *r* = 0.76) and relative REM duration (*p* < 0.01; *r* = 0.77), with higher verbal fluency score spent more time in relative S3 duration (*p* < 0.001; *r* = 0.7) and relative REM duration (*p* < 0.001; *r* = 0.72), with higher language score spent more time in relative S3 duration (*p* = 0.005; *r* = 0.5) and relative REM duration (*p* = 0.004; *r* = 0.51), with higher visuospatial abilities scores spent more time in relative S3 duration (*p* < 0.001; *r* = 0.66) and relative REM duration (*p* < 0.001; *r* = 0.68). The significant positive correlation is shown in Figs. 1 and 2 respectively.

### Relationship between cortical volumetric measures and sleep macrostructure parameters

**Fig. 3:**
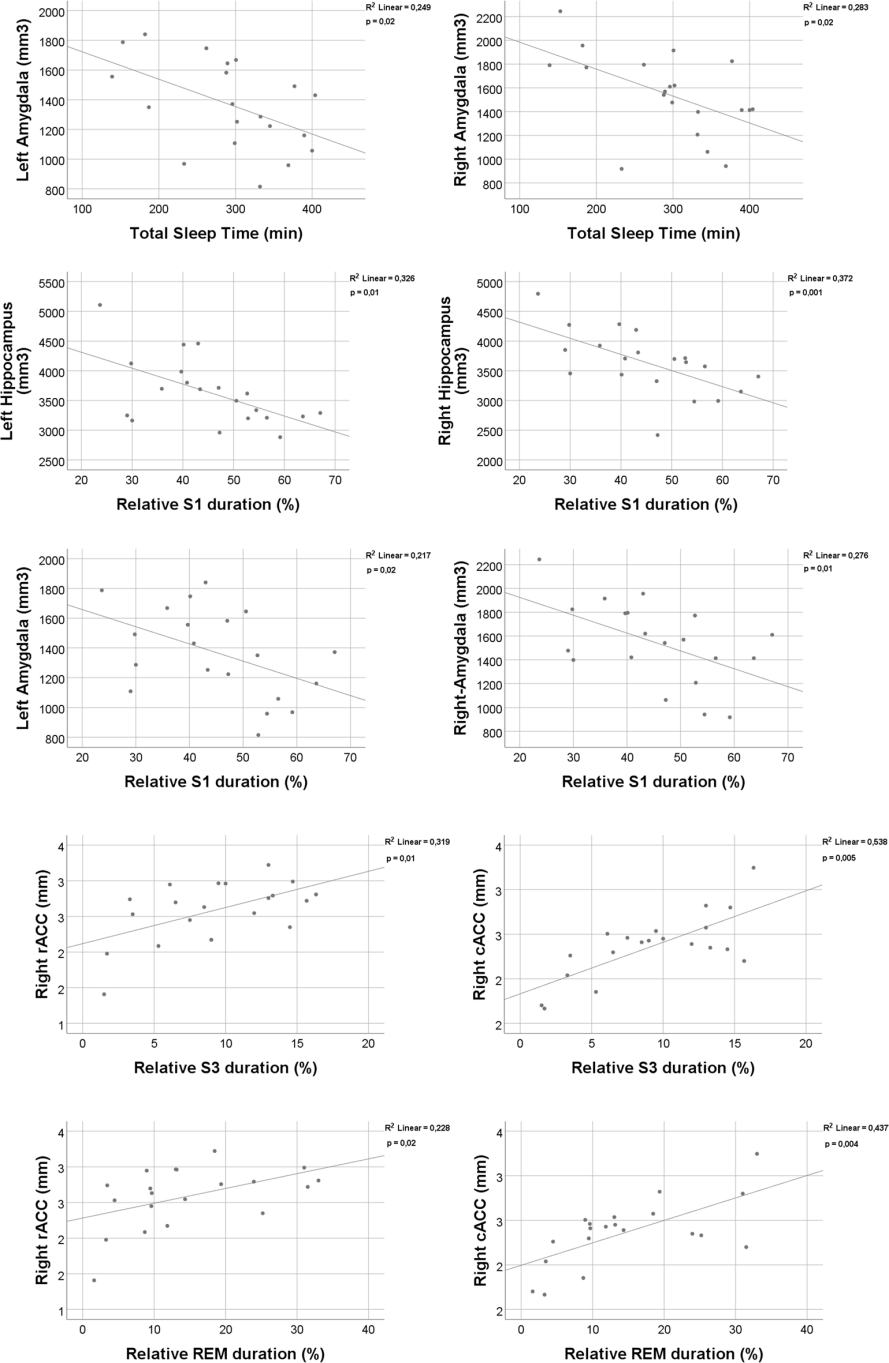
Correlation analysis between sleep macrostructure parameters and thickness and volume of the different cortical areas using Spearman correlation. Key: cAAC caudal anterior cingulate cortex, rACC rostral anterior cingulate cortex, REM rapid eye movements, S1 first stage of NREM, S3 third stage of NREM

Spearman’s correlation was applied to reveal the relationship between cortical volumes and sleep macrostructure parameters. All significant correlations are presented in Fig. 3. Subjects with decreased left and right amygdala volume spent more time sleeping (*p* = 0.02; *r* =  − 0,52; *p* = 0.02; *r* =  − 0.51) and in relative S1 sleep (*p* = 0.02; *r* =  − 0.53; *p* = 0.01; *r* =  − 0.56). Subjects with decreased left and right hippocampus volume also spent more time in S1 sleep (*p* = 0.01; *r* =  − 0.55; *p* = 0.001; *r* =  − 0.69). Subjects who spent more time in S2 sleep also showed decreased thickness of the right rostral anterior cingulate cortex (rACC) (*p* = 0.007; *r* =  − 0.58). Subjects who spent less time in S3 sleep also showed decreased thickness of right rACC (*p* = 0.01; *r* = 0.54), right caudal anterior cingulate cortex (cACC) (*p* = 0.005; *r* = 0.61) and with average cingulate cortex, which is the average cortical thickness of different parts of the cingulate cortex (*p* = 0.047; *r* = 0.45). Subjects who spent less time in REM showed decreased thickness of right rACC (*p* = 0.02; *r* = 0.53), right cACC (*p* = 0.004; *r* = 0.61), and average cingulate cortex (*p* = 0.048; *r* = 0.45). Subjects who had decreased REM latency showed decreased thickness of left cACC thickness (*p* = 0.015; *r* = 0.54).

### Relationship between Addenbrooke’s Cognitive Examination total score and sleep microstructure

**Fig. 4:**
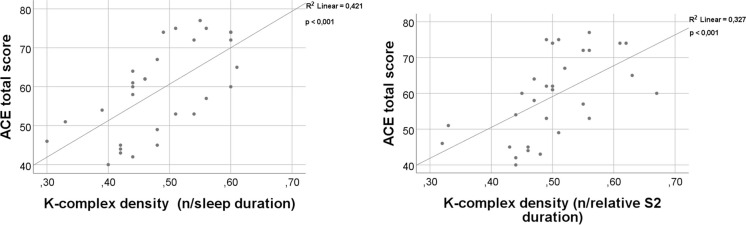
Correlation analysis between ACE total score and sleep microstructure parameters using Spearman correlation. Key: ACE Addenbrooke Cognitive Examination; S2 second stage of NREM

Spearman’s correlation was used to test the relationship between the different sleep microstructure parameters and ACE total scores. The ACE total score was higher in subjects with higher density of KC averaged for TST (*p* < 0.001; *r* = 0.66) and averaged for S2 sleep duration (*p* < 0.001; *r* = 0.64) (Fig. 4).

### Relationship between cortical measurements and sleep microstructure

**Fig. 5:**
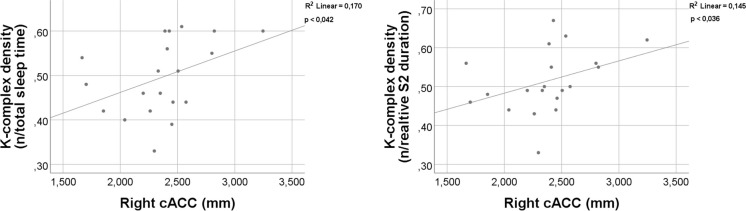
Correlation between K-complex density and the thickness of right caudal anterior cingulate cortex (cACC). Key: S2 second stage of NREM

Spearman’s correlation was applied to investigate the relationship between the cortical volumes and sleep microstructure parameters. Subjects with decreased right cACC thickness had decreased density of KC averaged for TST (*p* < 0.042; *r* = 0.458) and averaged for S2 sleep duration (*p* < 0.036; *r* = 0.472) (Fig. 5).

## Discussion

In our study, we compared KC density and the macrostructure of sleep of people with AD and healthy controls. Our results show increased sleep latency and decreased stage 3 duration parallel with increased superficial sleep (stage 1) duration in AD. The number of KCs were also decreased in people with AD. The decrease in KC density, REM sleep duration and stage 3 duration strongly correlated with deterioration of global cognitive functioning. The analysis of neuroimaging data revealed that the increase of superficial sleep associated with the atrophy of the mesio-temporal lobe structures, while the reduction of deep sleep and KCs correlates with the atrophy of the cingulate cortex.

Reduced sleep time and increased sleep latency are consistent findings of human sleep studies [49, 50] and in animal models [51] of AD. We reinforced these earlier results showing that people with AD sleep about 1 h less, and they have a ~ 30-min longer sleep latency than healthy controls. Some reports propose that decreased sleep duration and impaired sleep quality might associate with the presence of depressive symptoms which are frequently found among people with AD [52]. To eliminate this effect, our study did not include people with AD characterized by depressive symptoms or increased level of anxiety, showing probably more the individual effect of AD. The increased amyloid burden is also a factor for sleep loss [49, 53] with a bidirectional relationship where impaired sleep regulation also accelerates the amyloid-beta deposition rate [54]. It is confirmed by longitudinal observations reporting that sleep fragmentation associates with increased risk for the development of AD pathology, while intact sleep macrostructure signals low AD risk [55]. Animal models also reinforce the previous statements showing that sleep deprivation triggers impaired metabolism of tau [56]. Interestingly, AD people show various individual extent in the loss of deep sleep. A recent hallmark demonstrated that cognitive functions could be relatively stable over time in people with AD if sleep macrostructure changes are within a middle range [57], especially considering the relatively preserved REM sleep and SWS. Amyloid and tau AD models demonstrated similar patterns as animals showed different extent of contextual memory impairment, and amyloid/tau accumulation in line with the level of chronic sleep restriction and related circulating corticosterone level [58]. This association is confirmed directly by our findings indicating a strong correlation between better neuropsychological performance and less affected sleep macrostructure (largest *r* values are found between ACE total score and SWS/ REM sleep). The correlation suggests that intact sleep macrostructure might be beneficial in the maintenance of cognitive functioning validating the concept of antidementia trials targeting sleep changes [59, 60]. Furthermore, our results highlight that reduced sleep time strongly associates with poor global cognitive functioning, also showing that aiming to maintain relatively normal sleep time and to preserve deep sleep might be first-order priorities of therapeutic protocols in AD.

The association between high-quality sleep and preserved cognitive functioning might stand on two pillars. Firstly, SWS is a key factor in the elimination of amyloid-beta [54]. In a CSF study on healthy individuals, decreased slow-wave activity was strongly correlating with increased levels of amyloid-beta [61]. Other studies found that the described effect of SWS is independent from age or apoE status [25]. Two novel animal AD models might provide an ideal opportunity for the deeper understanding of this observation [62]. Similar effect of SWS has been found in relation to late-life tau burden as well [63]. While the exact physiology behind this phenomenon is not completely understood, recent animal studies indicate that loss of SWS is directly linked to the increased expression of cyclin-dependent kinase 5 (CDK5) as a result of decreased DNA methylation of CpG sites in the promoter region of CDK5 gene [64]. Besides the epigenetical modification, growing body of evidence indicates that the protective effect of SWS is also related to the association between amyloid clearance and the functions of glymphatic system of the brain [12]. The second pillar of the protective role of sleep is related to the memory consolidation process. For proper learning functions, the SWS-related synchronization between the hippocampus, thalamus, and neocortex is essential [65]. The highlighted physiological mechanism is crucial and probably specific for the mammalian brain leading to evolutional advantages as an extraordinary learning ability [66]. Previous reports highlighted that the accumulation of amyloid in the prefrontal cortex leads to impaired SWS-dependent thalamocortical coupling [53, 67, 68]. Furthermore, a prominent reduction of thalamic sleep spindles as key components of the process is a signature of AD [26], probably associated with the presence of tau pathology [69]. The malfunctioning of memory consolidation process is a common hallmark of all the animal models of AD independently from the genetic modification or pathology induction process [70–72]. Interestingly, the impaired hippocampal-cortical coupling is detectable even beside preserved hippocampal neural functioning [73]. Studies highlighted that beside the impairment of SWS, diffuse cortical amyloid deposition also associates with REM reduction [74]. However, in comparison with SWS, REM sleep interferes more with amyloid load probably due to the orexinergic mechanisms [75, 76] instead of the amyloid clearance, Nevertheless, proper REM sleep is crucial for spatial and contextual memory consolidation [77]. While the exact association between AD pathology and SWS/REM sleep is still not completely understood, the link between the preservation of these sleep stages and proper cognitive functioning is well established and further supported by our correlation analysis (Figs. 1 and 2). Thus, both REM and SWS could serve as a promising intervention target for AD.

The described characteristics of sleep macrostructure changes in AD (Table 2) are in line with the previous literature. SWS is diminished in people with AD, REM duration is reduced but in a lesser extent, while REM latency is preserved [78, 79]. Unfortunately, low number of transgenic animal studies analysed the above-described pattern; however, similar observations have been indicated [80, 81]. According to the current concept, the global loss of SWS becomes dominant once tau pathology reaches the cortex in MCI stage [38, 82]. The link between cortical involvement and the decrease of SWS/ REM sleep duration might be explained by previous neuroanatomical studies showing the dominance of cingulate cortex, orbitofrontal cortex, and precuneus in the generation of SWS activity [83] and the role of anterior cingulate cortex and parietal operculum in the generation of REM sleep [84]. Thus, disruption of SWS and REM sleep depends mostly on the atrophy of cortical areas instead of subcortical structures as hippocampus or amygdala [85]. Our results support this concept showing that SWS/REM duration changes correlate only with atrophy of cortical areas, with the atrophy of ACC (Fig. 3). The strong correlation between superficial sleep loss and smaller hippocampal/amygdaloid volumes might be explained by the disease course of AD. Mesio-temporal atrophy is present even in the preclinical stages of AD [86, 87] due to the accumulation pattern of amyloid. Deposition of amyloid in the entorhinal regions precedes the appearance in the cingulate cortex [88]. Thus, the early changes in sleep structure like the increase of fragmented superficial sleep occur simultaneously with the entorhinal neurodegeneration [89, 90], and the correlation remains significant and robust through the entire disease course. Another possible explanation is that neurons of the hippocampi and amygdaloid bodies show the highest vulnerability for degeneration to sleep loss [90, 91], pointing to the preventive role of sleep in the pathomechanism of AD. While the entire connection between sleep pattern and neuroanatomical disease course is barely understood, sleep fragmentation seems to be an early preclinical marker, while the appearance of SWS/ REM loss is indicative for the diminishment of long-range brain circuits, as a critical stage of AD progression [92].

Interestingly, while KC has been observed in cats [29] and rats as well [93], we completely lack studies on the relation of KC and AD animal models. Also, only a few human studies analysed the changes of KC density in AD and replication of earlier findings is lacking. Reda et al. did not find significant differences in KC density in people with MCI and healthy controls [39], proposing that KC reduction occurs in the later phase of the disease. However, two studies from 2020 observed a gradual decrease in KC density across healthy subjects, people with MCI, and AD [40]. Liu et al. suggested that KC reduction might signal the conversion of MCI into AD in a 2-year follow-up period [41]. A recent observation on 36 individuals reinforced the proposed concept showing that reduced KC density correlates with the conversion of amnestic-type MCI into AD dementia [94]. In the current study, we were able to replicate the findings of DeGennaro et al. using the same methodological approach for KC detection [38]. The previous study reported a 42.7% decrease of KCs in stage 2 in AD, while we found a 36.1% reduction in stage 2 KC density compared to healthy controls. Based on these findings, we can postulate that the decrease of KC indicates the transition from mild symptoms into severe presentation which potentially indicates the cortical involvement of neurodegenerative process. Thus, the reduction of KC might be a biomarker of AD pathology in MCI and could help to identify patients with the highest risk for the development of AD.

The above-mentioned concept is supported by the observation showing that a decrease in KC density associates with cortical atrophy in AD. Our observation points to the key link between the atrophy of ACC and the reduction of KC density (Fig. 5), which is in line with the current literature. KC is a characteristic event of non-REM sleep. The importance of AAC in the generation of non-REM sleep has been highlighted in EEG-fMRI co-recording studies [95]. Previous reports using magnetoencephalography also demonstrated that the generation of KC is highly dependent on the ACC functions [96]. Another study using electrical stimulation in patients with epilepsy surgeries marked the primary role of dorso-caudal ACC in the generation of KC [97]. Considering these results, significant decrease of KC density becomes evident as soon as the cingulate cortex is getting involved in the neurodegenerative process of AD pathology, which usually takes place in the late, transitional state of MCI [98, 99]. Thus, our findings reveal explanation for the previous observations showing that KC density might serve as a biomarker for the conversion of amnestic MCI into AD dementia [40, 41, 94]. Since cingulate cortex is one of the most preserved cortical areas across the mammalian species [100], more translational studies are needed on the underlying physiology of KC changes in AD pathology. The strong correlation between global cognitive performance and KC density (Fig. 4) draws the attention to the role of KC in proper cognitive functioning. Based on neurophysiological observations, KCs correspond to isolated and robust cortical down-states [35] which associate with the reduction of synaptic strength, counterbalancing the wakefulness attached synaptic enhancement [101, 102]. This process is crucial in the proper memory consolidation mechanism and in the accurate selection of relevant and irrelevant encoded memory items [103, 104]. Thus, it is intriguing to postulate two mechanisms: (1) the impaired sleep architecture and the fall of KC result in dysfunctional memory consolidation and cognitive function; and (2) the accelerated neurodegeneration process destroys the crucial cortical generators of sleep.

The current results should be cautiously considered, and some limitations must be addressed. The study has a relatively low sample size; however, it is the largest case–control pool for the analysis of KC in relation of AD. Furthermore, we did not perform follow-up for healthy controls; therefore, we do not know the clinical outcome of these participants regarding the potential development of MCI or AD. As a common limitation of sleep studies, we analysed the data obtained from one-night sleep recording which might not represent completely the sleep characteristic pattern of the examined individual.

In conclusion, analysis of sleep macro- and microarchitecture provides a unique window for the better understanding of the pathomechanism of cognitive decline. Our results highlight the crosstalk between cortical atrophy, sleep structure, and cognitive performance. The recent report reinforces the credibility of drug and non-pharmacological interventions targeting sleep as potential disease-modifying approaches in AD. Finally, our observation emphasizes the utility of quantitative KC assessment as a potential biomarker of the progression of AD.

## Clinical implication and future directions

### Implication of sleep as a biomarker

Since AD represents a progressive neurodegenerative disease, early detection, probably even in preclinical stage is crucial for successful interventions [105]. The most recent international diagnostic guideline put significant emphasis on the timely detection and recognition of prodromal stages [106]. As we proposed, sleep structure could be a significant component in a multimodal biomarker setting in screening and timely diagnosis. Reduced sleep quality and increased sleep fragmentation might serve as a potential prognostic marker indicating early amyloid pathology in preclinical stage and higher risk for the further development of AD dementia [107, 108]. It is easily and cost effectively measurable even in large populations using actigraphy [109]. While sleep quantity does not associate with increased conversion of MCI into dementia [110], additional markers might be beneficial. The reduction of KC, SWA, SWS, or sleep spindles is a prominent finding in MCI stage, where the neocortex becomes highly involved in toxic protein accumulation. Thus, these polysomnography parameters might be ideal candidates in MCI as red flag indicators for the conversion into dementia [41, 94, 111]. Since, KC is the less studied among these, and translational research is almost completely absent, human and animal observations are much needed on the relation of KC and cognitive decline. Furthermore, AD is a heterogeneous disorder with various phenotypes showing different neuroimaging profile, amyloid and tau accumulation pattern, clinical representation, progression rate, and therapy response [112]. Since analysis of sleep seems to be also promising in the biological phenotyping of AD variants [113], description of sleep pattern might facilitate personalized decisions to find the most optimal therapeutic regime for everyone affected by AD pathology [114]. For the proper use of sleep characteristics as a biomarker in AD, well-designed cross-correlation studies are needed on the link between sleep markers and established AD biomarkers.

### Implication of sleep as a therapeutic target and preventive strategy

As the Lancet Commission reports, approximately 40% of dementia cases could be prevented with timely initiated lifestyle interventions [115]. While sleep is not addressed as an individual risk factor in the statement of the commission, the strong link between cardiovascular health and sleep quality is well established. Reduced sleep duration and subjective sleep quality associate with elevated hazard ratios for coronary disease [116], while the presence of obstructive sleep apnoea is strongly connected to the higher risk of diabetes and hypertension [117]. Therefore, targeting sleep disturbances is a promising direction in disease modification and prevention of AD. Unfortunately, the current literature is not conclusive on the best strategies to modify sleep pattern [118]. As a meta-analysis highlighted, multi-modal intervention trials showed the most promising results in the improvement of sleep efficiency including light exposure, electrotherapy stimulation, physical exercise, and cognitive behavioural therapy [119]. Specifically targeting SWS seems to have superior effectiveness, where closed-loop acoustic stimulation is a promising approach [120]. Electrical stimulation, administration of interleukin-6, sodium oxybate, and tiagabine also improved SWS and, consecutively, cognitive functions [121]. To exploit the maximum benefit of multimodal interventions, randomized clinical trials are needed on the efficacy of sleep promotion in the prevention of cognitive deterioration [122].

### Implication of sleep as a marker of disease pathophysiology

As a relatively novel observation, amyloid and tau clearance follows a diurnal rhythm with strong fluctuations [123]. The diurnal fluctuation becomes highly attenuated following the amyloid plaque formation [124]. The regulation of the pattern significantly depends on the circadian clock [125], a mechanism highly affected by AD pathology [126], even in the preclinical stage [127]. Until now, we have relatively limited ability to analyse these changes in humans, since amyloid level was measurable for clinical use only from CSF [25, 54, 61, 63]. The recent guideline of the National Institute of Aging and Alzheimer’s Association recommends the use of blood amyloid markers equivalently with CSF [106], opening a new perspective on the understanding of diurnal pattern of amyloid. There is an urgent need for blood-based studies on the diurnal pattern of amyloid clearance to better understand the physiological process of amyloid accumulation and also its dependence on the individual circadian characteristics. There results might influence the field of blood-based diagnostics of neurodegenerative disorders and also will have an impact on the development of biological antibody therapies.

## Data Availability

The raw dataset is available upon reasonable request sent to the corresponding author.
